# Supplementary material for: Seeing an apocalyptic post-antibiotic future lowers antibiotics expectations and requests
Source: Commun Med (Lond). 2024 Jul 12;4:141. doi: 10.1038/s43856-024-00567-y (PMC11245540; doi:10.1038/s43856-024-00567-y)
Supplement: Supplementary file 2 — Supplementary Materials [file 43856_2024_567_MOESM2_ESM.pdf]

Supplementary Methods

Short Film

In the next page, you will be asked to watch a short film. Please, watch carefully the whole film.

[Participants watched either a film Catch, for more information see http://www.catchshortfilm.com/ or a film Digital Antiquities see more information https://itvs.org/films/digital-antiquities]

Viral Infection Scenario (Ear Infection)

Please read the text below carefully and imagine that the situation described is real.

In the last two days, you have experienced a lot of pain in your right ear. In addition, you felt pressure inside it and the skin around it was itchy and irritated. Since yesterday, the ear has felt blocked and you cannot hear properly. You feel feverish and when you checked you had a high temperature (around 39.0°C). The fever responds to ibuprofen but rises again after a few hours. You do not have a cough or breathing problems.

Today, as the earache is getting worse, you decide to see your GP. After taking your history and discussing your symptoms, she examines your ears and throat. Your GP tells you that it looks like you have a viral ear infection in your right ear. She explains that most ear infections clear up by themselves and that you should feel better in just a few days.

She recommends rest, drinking a lot of fluids, and taking painkillers such as ibuprofen to manage any pain and control the fever.

Dependent Variable: Expectations for antibiotics

In the situation described above...

❍ I should get a prescription for antibiotics.
❍ I should be offered a prescription for antibiotics.
❍ I would want my doctor to give me a prescription for antibiotics.
❍ I would not want my doctor to offer me a prescription for antibiotics.

Scale: Strongly disagree, Disagree, Mildly disagree, Mildly agree, Agree, Strongly agree

Dependent Variable: Likelihood of requesting antibiotics

In the situation described above...
❍ I would request a prescription for antibiotics.
❍ I would mention antibiotics to my doctor.
❍ I would suggest that I should have antibiotics.
❍ I would demand a prescription for antibiotics.

Scale: I certainly would not, I would not, I probably would not, I probably would, I would, I certainly would

Bacterial Kidney Infection Scenario

Please read the text below carefully and imagine that the situation described is real.

Yesterday you began to feel some discomfort in your side and lower back. After a few hours, this discomfort worsened and became very painful. You also have a high temperature (around 39.0°C) and feel the need to urinate often. When urinating, you experience a burning sensation and notice blood in your urine. You feel very weak and you have no appetite for food.

Today, as the symptoms have not improved, you have decided to see your GP. During the consultation, you discuss your symptoms, your previous illnesses and past medication usage. Your GP takes your temperature and checks your blood pressure before asking you to provide a sample of urine.

The urine test comes back positive for bacteria. Your GP tells you that the positive urine sample and your presenting symptoms mean that you have a bacterial kidney infection. She explains that kidney infections, caused by bacteria travelling from your bladder into one or both of your kidneys, require prompt treatment with antibiotics for symptom relief and to prevent serious complications.

She prescribes a 14-day course of antibiotics and some painkillers. She then mentions that you should start to feel better soon after taking the medication and the infection should have cleared up completely in about two weeks.

Dependent Variable: Adherence to a course of antibiotics

Would you take the 14-day course of prescribed antibiotics as recommended by your GP?
❍ Yes
❍ No

Follow-up questions

To what extent has the short film you have just watched affected your expectations for antibiotics?
- ❍ Not at all
- ❍
- ❍
- ❍
- ❍ Very much

Describe **briefly** what aspect of the short film you have watched affected your expectations for antibiotics.

Have you heard about "World Antibiotic Awareness Week" in November 2017?
- ❍ Yes
- ❍ No

Have you seen this short film on antibiotics called "Catch" (see screenshot above) **before** the experiment today?

[Participants were shown an image from a scene in the film, which we do not reproduce here for copyright reasons. The image depicts a young girl and her father, dressed in a protective suit, in a

room covered with plastic, suggesting quarantine conditions. They are sitting on the floor: the girl is looking at her plate full of food, while the man is watching her with concern. This scene is the same one that was used in the film’s promotional poster.]

Catch is a fictional short film about a father and daughter quarantined in their home in a post-antibiotic world.

- ❍ Yes
- ❍ No

Demographics

You have almost finished. Please answer the following questions.

What is your age? [dropdown options “18” years till “100 or more years”]

What is your gender?

- ❍ Male
- ❍ Female
- ❍ Other

Which of the following best describes your highest achieved education level?

- ❍ Less than High School
- ❍ Finished High School
- ❍ Undergraduate Degree
- ❍ Master's Degree
- ❍ Doctoral (PhD) or Professional (JD, MD) Degree
