# Supplementary material for: Seeing an apocalyptic post-antibiotic future lowers antibiotics expectations and requests
Source: Commun Med (Lond). 2024 Jul 12;4:141. doi: 10.1038/s43856-024-00567-y (PMC11245540; doi:10.1038/s43856-024-00567-y)
Supplement: Supplementary file 3 — Reporting Summary [file 43856_2024_567_MOESM3_ESM.pdf]

nature portfolio

Corresponding author(s): Miroslav Sirota

Last updated by author(s): Apr 26, 2023

Reporting Summary

Nature Portfolio wishes to improve the reproducibility of the work that we publish. This form provides structure for consistency and transparency in reporting. For further information on Nature Portfolio policies, see our Editorial Policies and the Editorial Policy Checklist.

Statistics

For all statistical analyses, confirm that the following items are present in the figure legend, table legend, main text, or Methods section.

n/a	Confirmed	
☐	☒	The exact sample size (n) for each experimental group/condition, given as a discrete number and unit of measurement
☐	☒	A statement on whether measurements were taken from distinct samples or whether the same sample was measured repeatedly
☐	☒	The statistical test(s) used AND whether they are one- or two-sided<br>*Only common tests should be described solely by name; describe more complex techniques in the Methods section.*
☐	☒	A description of all covariates tested
☐	☒	A description of any assumptions or corrections, such as tests of normality and adjustment for multiple comparisons
☐	☒	A full description of the statistical parameters including central tendency (e.g. means) or other basic estimates (e.g. regression coefficient) AND variation (e.g. standard deviation) or associated estimates of uncertainty (e.g. confidence intervals)
☐	☒	For null hypothesis testing, the test statistic (e.g. F, t, r) with confidence intervals, effect sizes, degrees of freedom and P value noted<br>*Give P values as exact values whenever suitable.*
☐	☒	For Bayesian analysis, information on the choice of priors and Markov chain Monte Carlo settings
☒	☐	For hierarchical and complex designs, identification of the appropriate level for tests and full reporting of outcomes
☐	☒	Estimates of effect sizes (e.g. Cohen's d, Pearson's r), indicating how they were calculated

Our web collection on statistics for biologists contains articles on many of the points above.

Software and code

Policy information about availability of computer code

Data collection	Data collection was completed using Qualtrics survey software.
Data analysis	Data analysis was conducted in R 4.1.2.

For manuscripts utilizing custom algorithms or software that are central to the research but not yet described in published literature, software must be made available to editors and reviewers. We strongly encourage code deposition in a community repository (e.g. GitHub). See the Nature Portfolio guidelines for submitting code & software for further information.

Data

Policy information about availability of data

All manuscripts must include a data availability statement. This statement should provide the following information, where applicable:

- Accession codes, unique identifiers, or web links for publicly available datasets
- A description of any restrictions on data availability
- For clinical datasets or third party data, please ensure that the statement adheres to our policy

Data are publicly available at https://osf.io/rmvck/

Human research participants

Policy information about studies involving human research participants and Sex and Gender in Research.

Reporting on sex and gender	73.0% of our sample has identified as female, 26.5% as men and 0.5% selected another option. We did not collect information on sex. We did not conduct statistical analyses testing a possible moderation effect of gender on the intervention effect. This is because we did not collect an appropriate balanced sample to test the moderation effect of this variable.
Population characteristics	Please see below.
Recruitment	Participants in the experiment were recruited via SONA or via snowballing technique. Participation was voluntary, self-selected and participants could quit the questionnaire at any time. Therefore, the studies might suffer from a self-selection bias. The individuals who were more interested in the topic of health and antibiotics might be over-represented. Since individuals were randomly assigned to the experimental conditions, our causal inferences should not be affected by this bias.
Ethics oversight	The Ethics Committee of the Department of Psychology, University of Essex, has approved the study protocol.

Note that full information on the approval of the study protocol must also be provided in the manuscript.

Field-specific reporting

Please select the one below that is the best fit for your research. If you are not sure, read the appropriate sections before making your selection.

☐ Life sciences ☒ Behavioural & social sciences ☐ Ecological, evolutionary & environmental sciences

For a reference copy of the document with all sections, see nature.com/documents/nr-reporting-summary-flat.pdf

Behavioural & social sciences study design

All studies must disclose on these points even when the disclosure is negative.

Study description	The study was a quantitative experiment containing three waves of participants designed to test the effect of a film intervention relative to a baseline condition.
Research sample	The participants were members of the public and students recruited locally. The analytical sample consisted of 378 participants (ages ranging from 18 to 59 years, M = 22.6, SD = 7.0 years; 73.0% of whom identified as female, 26.5% as men and 0.5% selected another option). The levels of the participants' education were relatively heterogeneous: 0.3% did not complete their high school education, 59.9% completed high school education, 36.8% completed a college degree, 3.7% completed a master's degree and 0.3% completed a PhD or other professional degree. This sample is not a representative sample of the UK population.
Sampling strategy	We used a convenience sampling strategy. The sample size was determined by a-priori power calculations to test each hypothesis. We aimed to recruit 128 participants in each of the three waves based on an a-priori power analysis to be able to detect a medium effect size (Cohen's $d = 0.5$) assuming $\alpha = .05$ and $1 - \beta = .80$ for an independent-samples t-test. The sampling and recruitment is detailed in the Methods section.
Data collection	Data from the experiment were collected in lab or online using Qualtrics survey software. All randomisations were conducted automatically by the default randomisation algorithm embedded in Qualtrics. Thus, the researchers and participants were blinded to the condition allocation process.
Timing	We collected data from the different groups of UK participants, each at a different point in time (i.e., waves). Wave 1 was the Pre-COVID wave, from 13/2/2018 to 16/03/2018, before the pandemic. Wave 2 was the COVID lockdown wave from 24/2/2021 to 28/5/2021, during and shortly after the third and last lockdown which ended on 29 March in the UK. Wave 3 was the COVID post-lockdown wave from 11/11/2021 to 02/5/2022, when restrictions were gradually lifted despite the spread of the Omicron variant.
Data exclusions	Following the a-priori exclusion criterion, 11 participants were excluded because they failed to watch the video fully (i.e., less than 825 seconds in the control condition and less than 910 seconds in the intervention condition).
Non-participation	Unfortunately, it is not possible to estimate the response rate because of self-selection of the participants and snowballing technique. We recorded 432 attempts to complete the questionnaire; 389 participants completed the questionnaire (dropout: n = 43, i.e., 10% out of the overall sample).
Randomization	Participants were randomly allocated to the experimental conditions. The random allocation of the participants was done by the Qualtrics built-in randomiser, which operates automatically using the Mersenne Twister algorithm. Thus, the researchers were blinded to the condition allocation process.

Reporting for specific materials, systems and methods

We require information from authors about some types of materials, experimental systems and methods used in many studies. Here, indicate whether each material, system or method listed is relevant to your study. If you are not sure if a list item applies to your research, read the appropriate section before selecting a response.

Materials & experimental systems

n/a	Involved in the study
☒	☐ Antibodies
☒	☐ Eukaryotic cell lines
☒	☐ Palaeontology and archaeology
☒	☐ Animals and other organisms
☒	☐ Clinical data
☒	☐ Dual use research of concern

Methods

n/a	Involved in the study
☒	☐ ChIP-seq
☒	☐ Flow cytometry
☒	☐ MRI-based neuroimaging
